# Watching TV has a distinct sociodemographic and lifestyle profile compared with other sedentary behaviors: A nationwide population-based study

**DOI:** 10.1371/journal.pone.0188836

**Published:** 2017-12-05

**Authors:** Elena Andrade-Gómez, Esther García-Esquinas, Rosario Ortolá, David Martínez-Gómez, Fernando Rodríguez-Artalejo

**Affiliations:** 1 Department of Preventive Medicine and Public Health, Universidad Autónoma de Madrid/ IdiPaz and CIBER of Epidemiology and Public Health (CIBERESP), Madrid, Spain; 2 Department of Physical Education, Sport and Human Movement, School of Teacher Training and Education, Universidad Autónoma de Madrid, Madrid, Spain; 3 IMDEA-Food Institute and CEI UAM+CSIC, Madrid, Spain; Universitat de Valencia, SPAIN

## Abstract

Watching TV has been consistently associated with higher risk of adverse health outcomes, but the effect of other sedentary behaviors (SB) is uncertain. Potential explanations are that watching TV is not a marker of a broader sedentary pattern and that each SB reflects different sociodemographic and health characteristics. Data were taken form a survey on 10,199 individuals, representative of the Spanish population aged ≥18 years. SB and other health behaviors were ascertained using validated questionnaires. Watching TV was the predominant SB (45.4% of the total sitting time), followed by sitting at the computer (22.7%). TV watching time showed no correlation with total time on other SB (r: -0.02, p = 0.07). By contrast, time spent at the computer was directly correlated with time spent on commuting (r: 0.07, p<0.01), listening to music (r: 0.10, p<0.01) and reading (r: 0.08, p<0.01). TV watching time was greater in those with older age, lower education, unhealthier lifestyle, and with diabetes or osteomuscular disease. More time spent at the computer or in commuting was linked to younger age, male gender, higher education and having a sedentary job. In conclusion, watching TV is not correlated with other SB and shows a distinct demographic and lifestyle profile.

## Introduction

Sedentary behaviors (SBs) are those waking activities characterized by low energy expenditure (≤1.5 metabolic equivalents, METs) that are performed in a sitting or reclining position [1]. Among the most frequent SBs, watching TV has been consistently associated with higher risk of several adverse health outcomes, independently of physical activity (PA) [2–8], but results on the association of other SB with health have been less consistent [9–14]. This might be due to several explanations. First, although watching TV and other screens is the predominant leisure-time SB [15], TV watching time might not be a marker of a broader sedentary pattern. For instance, in a sample of the population of urban areas of Adelaide in Australia, time spent watching TV was associated positively with time in other SB and negatively with leisure-time PA in women, but no such associations were observed in men [16].

Another potential explanation is that different SB may have different health effects. It has been suggested that TV and other “passive” SB including listening or talking while sitting, and sitting around could be more harmful than other “mentally-active” SB, such as computer-use and reading books or newspapers [17]. In fact, several studies have found that TV watching time, but not other SB (e.g. time seated at the computer, reading or commuting), is associated with cardio-metabolic biomarkers [9, 10], poor cognitive performance [11], and all-cause mortality [12]. Also, some studies have found a stronger association of metabolic syndrome, obesity and diabetes risk with time spent watching TV than with time spent seated in other activities, including at work or away from home or driving [4, 13, 14]. These apparently different associations of each SB may reflect that TV watching time is the predominant SB and is better recalled than time spent in other SB [18], but it is also possible that they partly result from distinct demographic and health characteristics of individuals with each SB, which might be difficult to account for in statistical analyses.

To our knowledge, no previous study on a representative sample of a whole country has examined the association between TV watching time and the rest of SB, or has reported the full profile of sociodemographic, lifestyle and health variables associated with each type of SB. Accordingly, the objective of this manuscript was to assess the correlation between time spent in different types of SB, as well as to identify the variables associated with each type of SB, in the adult population of Spain.

## Material and methods

### Study design and participants

Data were taken from the Study on Nutrition and Cardiovascular Risk in Spain (ENRICA), whose methods have been reported elsewhere [19, 20]. In brief, this was a cross-sectional study conducted between June 2008 and October 2010 with a representative sample of the non-institutionalized population of Spain aged 18 years and older. Participants were selected by stratified cluster sampling. First, the sample was stratified by province and size of municipality. Second, clusters were selected randomly in two stages: municipalities and census sections. Finally, the households within each section were selected by random phone dialing; participants in the households were selected proportionally to the sex and age distribution of the Spanish population.

Trained and certified staff collected information in three stages: a phone interview and two subsequent home visits. The phone interview obtained data on sociodemographic factors, health behaviors, self-rated health and morbidity. In the first home visit, blood and urine samples were collected and sent to a central laboratory for analytical determinations; and in the second visit, an electronic dietary history was obtained and a physical examination was performed. A total of 22,387 subjects were invited to participate in the study and 12,985 (58%) responded to the telephone interview. Of these, 12,880 (99.2%) provided a sample of blood and urine. Of these, 11,191 (86.9%) participated in the physical examination and provided dietary information. Therefore, the final response rate in the study was 51%. From the study participants, we excluded 992 without complete data on study variables; thus, the analytical sample included 10,199 (5,459 women, 4,740 men) individuals.

The Clinical Research Ethics Committee of ‘La Paz’ University Hospital in Madrid approved the study, and participants provided written informed consent.

## Study variables

### Sedentary behaviors

SB were ascertained with the questionnaire of the Nurses’ Health Study (NHS) validated in Spain [21]. Individuals reported the number of hours/week in the preceding year spent in six sedentary activities during leisure time: seated watching TV, seated while commuting, seated at the computer, seated or lying in the sun in summer and winter, seated or lying while listening to music (except in transportation), and seated while reading (except in transportation).

### Other variables

Study participants reported their sex, age, educational level (≤primary, secondary, and university studies), employment status (employed, not employed), and tobacco consumption (current, former and never smoker). Food consumption was obtained with a validated computerized diet history, developed from that used in the EPIC-cohort in Spain [22]. This diet history collected information on 34 alcoholic beverages and used photographs to help quantify portion sizes; this information served to classify study participants as non-drinkers (including also occasional drinkers), ex-drinkers, moderate drinkers, and heavy drinkers; the threshold between moderate and heavy intake was 40 g/day in men and 24 g/day in women [23, 24]. Participants were also classified according to their adherence to the Mediterranean Drinking Pattern (MDP), defined as moderate average alcohol consumption with wine preference and intake drinking only with meals [23, 24]. Finally, adherence to the Mediterranean diet was summarized using the MEDAS index [25]; a higher score on MEDAS (range 0–14) represented a better adherence.

Physical activity was assessed with the validated EPIC-Spain cohort questionnaire [26] and summarized according to the Cambridge Physical Activity Index [27]. This index includes four categories (inactive, moderately inactive, moderately active and active), which result from combinations of categories of physical activity at work and of duration (h/week) of physical activity at leisure (cycling, running, aerobics, swimming, etc.). Physical activity at work was obtained in five categories (sedentary occupation, standing occupation, manual, heavy manual work, and no work), which were grouped into sedentary and non-sedentary occupation. Recreational physical activity was expressed in MET-hour/day from walking, cycling and other types of exercise (running, soccer, aerobics, swimming, tennis, gymnastics), and was classified into tertiles. In addition, habitual light intensity physical activity during leisure time was estimated from the time devoted to household chores (cleaning, washing, cooking, taking care of children, etc.) and to gardening and do-it-yourself activities [26].

Weight and height were measured at home twice using electronic scales and portable extendable stadiometers. Mean values of the two measurements were used for analyses. Body mass index (BMI) was calculated as weight in kg divided by squared height in m. Normal weight was defined as a BMI<25 kg/m^2^, overweight as BMI 25–29.9 kg/m^2^, and obesity as BMI ≥30 kg/m^2^.

We also ascertained the time spent sleeping with the following questions: 1)“Can you tell me approximately how long you usually sleep at night?” and 2)“Can you tell me approximately how long you usually sleep during the day?” Participants were asked to report the number of hours and minutes they slept [28]. Lastly, study participants reported the following physician-diagnosed diseases: cardiovascular disease (ischemic heart disease, stroke, and heart failure), diabetes and osteomuscular disease (hip or knee osteoarthritis, arthritis).

### Statistical analysis

To assess the correlation between SB, we calculated partial Pearson correlation coefficients (r) adjusted for sex, age (continuous), education (≤primary, secondary, university studies), and employment status (employed, not employed). Given that BMI may confound the correlation between SB, we ran additional analyses with further adjustment for BMI.

Next, to identify the sociodemographic, lifestyle and clinical variables associated with each SB, we used linear regression models that were adjusted for sex, age (continuous), education (≤primary, secondary, university studies), and employment status (employed, not employed). The study associations were summarized with beta regression coefficients and their corresponding 95% confidence interval. For ordinal variables, we tested the dose-response relationship with P-values for trend, which were calculated by assigning a progressively increasing value (1, 2, 3) to each of the categories, and modeling them as a continuous variable. Finally we examined if the variables associated with each SB varied by sex and age; for this purpose, we used factorial F-tests that compared models with and without interaction terms (products of age or sex categories by the study variables). Given that in most cases P-values were >0.05 and that results were always similar in each sex and age group, study findings are presented for the total study sample.

Analyses were weighted to reconstruct the Spanish population, and were performed with the *survey* procedure in STATA (version 13.0, College Station, TX: StataCorp LP).

## Results

Watching TV was the predominant SB (45.4% of the total sitting time) among study participants, followed by being seated at the computer (22.7%), reading (15.3%) and commuting (11.8%) (Table 1).

**Table 1:** Time spent in sedentary behaviors (excluding at work) in the adult population of spain (ENRICA study, N = 10,199).

	Mean (SD), h/day	%
Watching TV	1.96 (1.40)	45.4
Using computer	0.98 (1.46)	22.7
Commuting	0.51 (0.59)	11.8
Lying in the sun	0.02 (0.14)	0.5
Listening to music^a^	0.19 (0.51)	4.4
Reading^a^	0.66 (0.85)	15.3

SD: Standard deviation

^a^ Except in transportation

Percentages do not sum 100 because of rounding

Table 2 presents the correlations between SB. TV watching time showed no correlation with total time spent in other SB (r: -0.02, p = 0.07), and showed a weak inverse correlation with the time being seated while commuting (r:-0.02, p = 0.05) and reading (r: -0.04, p<0.01). By contrast, it also showed a weak direct correlation with listening to music (r: 0.02, p = 0.03). However, time seated at the computer was directly correlated with time spent in commuting (r: 0.07, p<0.01), listening to music (r: 0.14, p<0.01) and reading (r: 0.11, p<0.01). Also, being seated or lying in the sun was directly correlated with listening to music (r: 0.06, p<0.01), and longer time listening to music was linked to longer time reading (r: 0.12, p<001). Results did not materially change after additional adjustment for BMI (data not shown in tables).

**Table 2:** Correlations (P-value) between the main sedentary behaviors in the adult population of Spain (ENRICA study, N = 10,199).

	Sedentary behaviors						
	Watching TV (h/day)	Other sedentary behaviors (h/day)^a^^,^*	Using computer (h/day)	Commuting (h/day)	Lying in the sun (h/day)	Listening to music (h/day)	Reading (h/day)
Watching TV (h/day)	1						
**Other sedentary behaviors (h/day)**^**a**^	-0.02 (0.07)	1					
Using computer (h/day)	0.00 (1.00)	0.13 (**<0.01**)*	1				
Commuting (h/day)	-0.02 (0.05)	0.07 (**<0.01**)*	0.07 (**<0.01**)	1			
Lying in the sun (h/day)	0.01 (0.60)	0.04 (**<0.01**)*	0.02 (0.06)	0.02 (0.11)	1		
Listening to music (h/day)	0.02 (**0.03**)	0.14 (**<0.01**)*	0.10 (**<0.01**)	0.02 (**0.02**)	0.06 (**<0.01**)	1	
Reading (h/day)	-0.04 (**<0.01**)	0.11 (**<0.01**)*	0.08 (**<0.01**)	0.01 (0.28)	0.02 (**0.02**)	0.12 (**<0.01**)	1

^a^ Includes sitting time at the computer, commuting, lying in the sun, listening to music, and reading.

*Not includes the sedentary behavior of interest.

Results are adjusted for sex, age (continuous), educational level (≤primary, secondary, university), and employment status (employed, not employed).

P-values <0.05 are presented in bold.

Table 3 shows the main variables associated with each SB. Watching TV time was greater in those with older age, lower education and unhealthier lifestyle (smoking, worse diet, less recreational physical activity, higher BMI), and in those with diabetes or osteomuscular disease. However, more time seated at the computer and in commuting was linked to younger age, male gender, higher education, and having a sedentary job. Other variables, including diet quality, recreational physical activity, household light intensity activity or night-time sleep were statistically linked to time seated at the computer or during commuting, but the associations were very weak. Associations were less marked for the other SB, but reading time was longer in older people, with higher education, who did more recreational physical activity, devoted less time to household chores and suffered from cardiovascular disease.

**Table 3:** Beta regression coefficients (95% confidence interval) for the association of sociodemographic factors, lifestyle and morbidity with time spent in sedentary behaviors in the adult population of Spain (ENRICA study, N = 10,199).

	Sedentary behaviors (h/day)					
	Watching TV	At the computer	Commuting	Lying in the sun	Listening to music ^a^	Reading ^a^
**Sociodemographic factors**						
Sex						
Men	Ref.	Ref.	Ref.	Ref.	Ref.	Ref.
Women	-0.01 (-0.08;0.06)	**-0.33 (-0.40;-0.26)**	**-0.22 (-0.25;-0.20)**	0.00 (-0.01;0.01)	**-0.10 (-0.13;-0.07)**	-0.04 (-0.08;0.00)
Age, years						
18–44	Ref.	Ref.	Ref.	Ref.	Ref.	Ref.
45–64	**0.30 (0.23;0.37)**	**-0.32 (-0.40;-0.23)**	**-0.18 (-0.22;-0.15)**	0.00 (-0.01; 0.01)	**-0.06 (-0.09;-0.03)**	**0.14 (0.10;0.19)**
≥65	**0.89 (0.77;1.00)**	**-0.68 (-0.76;-0.60)**	**-0.34 (-0.37;-0.30)**	0.00 (-0.01;0.01)	-0.01 (-0.05;0.04)	**0.33 (0.26;0.40)**
*P-trend*	**<0.01**	**<0.01**	**<0.01**	0.72	<0.01	**<0.01**
Educational level						
≤Primary	Ref.	Ref.	Ref.	Ref.	Ref.	Ref.
Secondary	**-0.29 (-0.38;-0.20)**	**0.43 (0.36;0.51)**	**0.06 (0.03;0.09)**	0.00 (-0.01;0.01)	0.02 (-0.01;0.05)	**0.34 (0.29;0.38)**
University	**-0.67 (-0.76;-0.58)**	**0.98 (0.89;1.08)**	**0.10 (0.07;0.14)**	0.00 (-0.01;0.01)	0.01 (-0.02;0.05)	**0.54 (0.48;0.59)**
*P-trend*	**<0.01**	**<0.01**	**<0.01**	0.65	0.55	**<0.01**
**Lifestyle**						
Smoking						
Current	Ref.	Ref.	Ref.	Ref.	Ref.	Ref.
Former	-0.19 (-0.28;-0.10)	0.07 (-0.02;0.17)	-0.02 (-0.06;0.01)	0.01 (-0.01;0.02)	-0.01 (-0.04;0.03)	0.01 (-0.05;0.07)
Never	-0.21 (-0.28;-0.14)	0.05 (-0.03;0.13)	-0.02 (-0.05;0.02)	0.00 (-0.01; 0.00)	0.00 (-0.03;0.03)	-0.02 (-0.08;0.03)
*P-trend*	**<0.01**	0.26	0.38	0.20	0.94	0.36
Alcohol intake^b^						
Non-drinker	Ref.	Ref.	Ref.	Ref.	Ref.	Ref.
Ex-drinker	0.17 (0.00;0.34)	0.01 (-0.12;0.13)	-0.01 (-0.07;0.05)	0.02 (0.00;0.04)	0.00 (-0.05;0.05)	-0.01 (-0.10;0.09)
Moderate drinker	-0.04 (-0.10;0.03)	0.03 (-0.04;0.10)	-0.01 (-0.04;0.02)	0.01 (0.00;.0.01)	-0.01 (-0.03;0.02)	-0.01 (-0.05;0.04)
Heavy drinker	0.08 (-0.06;0.22)	-0.07 (-0.21;0.07)	-0.07 (-0.12;-0.02)	-0.01 (-0.02;0.00)	0.03 (-0.03;0.08)	-0.04 (-0.12;0.04)
*P-trend* *(excluding ex-drinkers)*	0.66	0.87	0.13	0.52	0.98	0.50
Mediterranean drinking pattern (MDP)						
Non-drinker	Ref.	Ref.	Ref.	Ref.	Ref.	Ref.
Ex-drinker	**0.17 (0.00;0.33)**	0.01 (-0.12;0.13)	-0.01 (-0.07;0.05)	0.02 (0.00;0.04)	0.00 (-0.05;0.05)	-0.01 (-0.10;0.09)
Drinker with no MDP	-0.01 (-0.08;0.06)	0.02 (-0.06;0.10)	-0.02 (-0.05;0.01)	0.00 (-0.01;0.01)	0.00 (-0.03;0.03)	0.00 (-0.05;0.04)
Drinker with MDP	-0.07 (-0.17;0.03)	0.02 (-0.07;0.12)	0.01 (-0.03;0.04)	0.01 (0.00;0.02)	-0.01 (-0.04;0.03)	-0.03 (-0.09;0.02)
MEDAS score (tertiles)^c^						
≤6	Ref.	Ref.	Ref.	Ref.	Ref.	Ref.
7–8	**-0.09 (-0.17;-0.01)**	0.04 (-0.05;0.12)	0.00 (-0.04;0.03)	-0.01 (-0.01;0.00)	-0.02 (-0.04;0.01)	0.00 (-0.05;0.05)
≥9	**-0.19 (-0.26;-0.11)**	-0.02 (-0.09;0.06)	**-0.04 (-0.07;-0.01)**	-0.01 (-0.01;0.00)	-0.01 (-0.04;0.02)	0.01 (-0.03;0.06)
*P-trend*	**<0.01**	0.80	**0.03**	0.06	0.47	0.64
Cambridge's physical activity index						
Inactive	Ref.	Ref.	Ref.	Ref.	Ref.	Ref.
Moderately inactive	**-0.29 (-0.38;-0.21)**	**-0.22 (-0.31;-0.14)**	**0.04 (0.01;0.07)**	0.00 (-0.01;0.01)	-0.03 (-0.06;0.00)	0.03 (-0.03;0.08)
Moderately active	**-0.35 (-0.44;-0.26)**	**-0.27 (-0.37;-0.17)**	0.03 (0.00;0.07)	0.01 (0.00;0.02)	-0.01 (-0.05;0.02)	0.01 (-0.05;0.06)
Active	**-0.43 (-0.53;-0.33)**	**-0.42 (-0.53;-0.30)**	0.03 (-0.02;0.09)	**0.02 (0.01;0.03)**	-0.01 (-0.06;0.03)	0.03 (-0.04:0.09)
*P-trend*	**<0.01**	**<0.01**	0.18	**<0.01**	0.66	0.58
Sedentary work						
No	Ref.	Ref.	Ref.	Ref.	Ref.	Ref.
Yes	**-**0.06 (-0.12;0.01)	**0.78 (0.68;0.87)**	**0.05 (0.01;0.08)**	0.00 (-0.01;0.01)	0.01 (-0.02;0.04)	0.00 (-0.05;0.05)
Recreational physical activity (MET*h/week)						
≤18	Ref.	Ref.	Ref.	Ref.	Ref.	Ref.
>18-≤39	**-0.15 (-0.22;-0.07)**	0.02 (-0.06;0.09)	0.00 (-0.03;0.03)	0.01 (0.00;0.02)	0.04 (0.01;0.06)	0.08 (0.03;0.13)
>39	**-0.19 (-0.27;-0.12)**	**0.09 (0.01;0.17)**	**-0.03 (-0.06;0.00)**	**0.02 (0.01;0.02)**	**0.05 (0.02;0.08)**	**0.22 (0.16;0.28)**
*P-trend*	**<0.01**	**0.03**	0.09	**<0.01**	**<0.01**	**<0.01**
Performing household chores (MET*h/day)						
<Median (<3.90)	Ref.	Ref.	Ref.	Ref.	Ref.	Ref.
≥Median (≥3.90)	-0.03 (-0.10;0.04)	**-0.11 (-0.19;-0.04)**	0.00 (-0.03;0.02)	0.00 (-0.01;0.01)	**-0.03 (-0.06;0.00)**	**-0.06 (-0.10;-0.01)**
*P-trend*	0.39	**<0.01**	0.80	0.82	**0.04**	**0.01**
Gardening/do-it-yourself (h/day)						
≤Median (≤0)	Ref.	Ref.	Ref.	Ref.	Ref.	Ref.
>Median (>0)	**-0.21 (-0.28;-0.14)**	-0.01 (-0.08;0.06)	**0.06 (0.03;0.09)**	0.00 (-0.01;0.01)	0.00 (-0.03;0.03)	-0.03 (-0.07;0.01)
*P-trend*	**<0.01**	0.80	**<0.01**	0.69	0.94	0.18
Body mass index (kg/m^2^)						
>25	Ref.	Ref.	Ref.	Ref.	Ref.	Ref.
25–29.9	**0.15 (0.08;0.22)**	-0.11 (-0.19;-0.03)	0.00 (-0.03;0.03)	0.00 (-0.01;0.00)	-0.02 (-0.05;0.01)	0.01 (-0.04;0.07)
≥30	**0.37 (0.28;0.46)**	-0.07 (-0.17;0.02)	0.03 (-0.01;0.06)	**-0.01 (-0.02;0.00)**	-0.02 (-0.06;0.01)	-0.04 (-0.10;0.01)
*P-trend*	**<0.01**	0.07	0.15	**0.01**	0.15	0.18
Day-time sleeping (h/day)						
<Median (<0.14)	Ref.	Ref.	Ref.	Ref.	Ref.	Ref.
≥Median (≥0.14)	**0.11 (0.05;0.17)**	-0.01 (-0.08;0.05)	0.01 (-0.02;0.03)	0.01 (0.00;0.01)	0.01 (-0.01;0.04)	-0.02 (-0.06;0.02)
*P-trend*	**<0.01**	0.73	0.52	0.10	0.25	0.38
Night-time sleeping (h/day)						
<Median (<7)	Ref.	Ref.	Ref.	Ref.	Ref.	Ref.
≥Median (≥7)	0.01 (-0.06;0.08)	**-0.07 (-0.14;0.00)**	**-0.07 (-0.10;-0.04)**	0.00 (-0.01;0.01)	-0.03 (-0.06;0.00)	-0.03 (-0.08;0.02)
*P-trend*	0.77	**0.04**	**<0.01**	0.62	0.05	0.19
**Morbidity**						
Cardiovascular disease^d^						
No	Ref.	Ref.	Ref.	Ref.	Ref.	Ref.
Yes	0.00 (-0.24;0.24)	0.12 (-0.06;0.31)	-0.03 (-0.09;0.03)	0.01 (-0.02;0.04)	-0.05 (-0.10;0.01)	**0.23 (0.05;0.41)**
Diabetes						
No	Ref.	Ref.	Ref.	Ref.	Ref.	Ref.
Yes	**0.21 (0.07;0.34)**	0.00 (-0.09;0.10)	0.02 (-0.03;0.07)	0.00 (-0.01;0.02)	0.05 (-0.01;0.11)	-0.05 (-0.13;0.03)
Osteomuscular disease^e^						
No	Ref.	Ref.	Ref.	Ref.	Ref.	Ref.
Yes	**0.25 (0.15;0.35)**	-0.03 (-0.10;0.04)	0.01 (-0.02;0.04)	0.01 (0.00;0.02)	**0.06 (0.03;0.10)**	-0.05 (-0.10;0.00)

Results are adjusted for sex, age (continuous), educational level (≤primary, secondary, university), and employment status (employed, not employed). However, results for Cambridge's physical activity index and sedentary work are only adjusted for age, sex and educational level because the definition of these variables included employment status.

^a^ Except in transportation.

^b^Threshold between moderate and excessive alcohol intake: 40 g/d in men and 24 g/d in women.

^c^Adherence to the Mediterranean diet (range 0–14).

^d^Ischemic heart disease, stroke, or heart failure

^e^Hip or knee osteoarthritis or arthritis.

Statistical significant results (p<0.05) are presented in bold.

## Discussion

Our results in the adult population of Spain show that watching TV has no association with total time spent on the rest of leisure-time SB, but has an inverse weak association with time devoted to commuting and reading. This suggests that people are partly substituting these specific SB for TV watching. Moreover, each type of SB has a distinct demographic and lifestyle profile; while time watching TV was greater in those with older age, lower education, unhealthy lifestyle and who suffered from chronic morbidity, a longer time spent seated at the computer or in commuting was linked to younger age, male gender, higher education and a sedentary job. This could have contributed to differences in health problems associated with TV watching versus other SB observed in several studies.

Our results on the lack of correlation between watching TV and total time spent on the rest SB are consistent with those obtained among middle-age men from an urban area in Australia [16]. However, in the latter study, watching TV was directly associated with other SB among women; as argued by the authors, women spend more time than men in home-related chores outside of work hours, so it is possible that differences in the ways that women and men use their non-working hours may influence the gender difference found in their study [16]. Our results, obtained in a whole country, show that, compared to men, women spent less time seated using the computer, in transportation and reading, but more time doing household chores (2.82 vs. 1.08 h/day); however, it has not precluded observing a null correlation between TV time and the rest of SB in each gender. Thus, it is possible that gender differences in this correlation are context-specific and they should be studied across countries, cultures, etc.

In addition, our study shows that “mentally-active” SB, including using the computer and reading, tend to cluster and, thus, confirm results of an exploratory factor analysis of data from a postal survey in Japanese older adults [17]. Also in line with this investigation [17], we found that a “passive” sedentary time, such as TV watching, was associated with less recreational physical activity and higher body weight, while time at the computer and reading were linked to more recreational physical activity but less light-intensity activity at home.

Our results on the variables associated with SB broadly concur with those from a review of 109 studies (83 of them were cross-sectional) published from 1982 to 2011 [29]. In this review, TV viewing time increased with age and BMI, decreased with educational level and leisure-time physical activity, and did not vary with gender; however results on the link between watching TV and smoking were mixed [29]. Like our study, this review provides evidence that computer use decreases with age and increases with educational level; however no association was found between computer use and leisure-time physical activity, and results were inconclusive about the association with gender and BMI [29]. Lastly, results in our study and in the review [29] do not support a relationship between any type of SB and alcohol consumption. Our study extends knowledge in this field by considering more types of SB than most previous research [29]; moreover, the associations between SB and certain lifestyles (e.g., drinking and dietary patterns, light intensity physical activity at home, sedentary work), which we assessed in our study, have been under-researched [29].

### Methodological aspects

Some methodological aspects warrant a comment. First, this study was cross-sectional, so no causal inferences can be made from the observed correlations and associations. Second, information on SB was self-reported. Sedentary time estimated with the Spanish version of the NHS questionnaire has shown a moderate validity against accelerometry; specifically, the Spearman correlation between the ratio of sedentary lifestyle to physical activity obtained through the questionnaire and the objective estimation (Triaxial Research Tracker) was -0.58 (95% confidence interval -0.75 to -0.33) [21]. However, given that objective measurement methods only estimate total sitting time, self-report is the only way to assess the different domains and types of activities that characterize each type of SB. Also, different SB assessed with this questionnaire have predicted obesity, diabetes and other adverse health outcomes in studies in the US and Spain [4, 12, 20]. Notwithstanding this, we acknowledge that the validity of each type of reported SB is unknown and that we cannot exclude that some SB were performed concurrently (e.g., lying in the sun and reading). Third, in some cases statistical significance was achieved for very weak, and possibly irrelevant, associations (e.g., diet quality and time spent in commuting), due to the large sample of the study; thus these statistically significant associations should not be over-interpreted. Fourth, different lifestyle and clinical profiles associated with TV watching versus using a computer could partly reflect the fact that time spent in the former was much greater than in the latter. Indeed, in a sensitivity analysis changing the thresholds of mentally-active sedentary time from 1 h/day to 3 h/day among older Japanese people, higher mentally-active sedentary time became associated with being overweight [17]. Also, too much computer use has been associated with overweight and physical inactivity in a study of 2,650 middle-aged Australian adults [30]. Thus, future research should examine if, regardless of the specific activity performed (e.g., reading, commuting, computer use), a very prolonged sitting time could be harmful to health. Fifth, although our study included many lifestyle and clinical variables, we did not assess cognitive, social, or environmental factors potentially associated with SB. Future investigations must consider these variables, because they could mix their health effects with those of SB, and because they can be well suited for targeted interventions to reduce SB. Sixth, as regards generalizability of results, Spain is suffering a hard economic crisis, which may have affected SB (e.g., a higher unemployment rate has surely reduced sedentary jobs and time spent commuting). Thus, results could have been somewhat different had the data been obtained during the hardest period of crisis (2011–2024). Lastly, results should be replicated in countries with different patterns of SB; for instance, studies should be conducted in areas with limited access to internet, which may limit time spent at the computer and modify the observed associations.

## Practical implications

Our findings have practical importance. First, given that TV watching is not correlated with total time spent in other SB, future research should assess the health effect of each type of SB separately. Second, because each type of SB shows a distinct sociodemographic, lifestyle and health profile, interventions to reduce each type of SB may need to be targeted to different population subgroups. An third, our research sheds some light on the optimal choice for intervening on SB: substituting SB with a different behavior that involves some type of physical activity (e.g., walking, swimming, laps), versus altering behavioral topography (e.g., from sitting to standing) while continuing with the original activity (e.g., standing while working) [31]. Despite the first alternative is behaviorally complex, the inverse association between TV viewing time and recreational physical activity provides some evidence of time displacement, and suggests that increasing physical activity may lead to reducing TV time. However, this seems not be the case for using the computer and reading because they show a direct association with recreational activity. By contrast, the fact that the time using the computer was greater in those with a sedentary job suggests that a postural change from sitting to standing could be a sensible intervention to reduce sedentary time while working at the computer (because most sedentary work currently requires a computer) [31, 32].

## Supporting information

S1 File Database. (DTA)
